# Neuroengineering Frontiers: A Selective Review of Neural Interfaces, Brain–Machine Interactions, and Artificial Intelligence in Neurodegenerative Diseases

**DOI:** 10.3390/app152111316

**Published:** 2025-10-22

**Authors:** Mutiyat Usman, Simachew Ashebir, Chioma Okey-Mbata, Yeoheung Yun, Seongtae Kim

**Affiliations:** 1 Department of Mathematics and Statistics, North Carolina A&T State University, Greensboro, NC 27411, USA; 2 Department of Biomedical Engineering, Applied Science and Technology, North Carolina A&T State University, Greensboro, NC 27411, USA; 3 Bioengineering Program, Department of Chemical, Biological, and Bioengineering, North Carolina A&T State University, Greensboro, NC 27411, USA

**Keywords:** brain–computer interfaces, co-adaptation, neurodegenerative diseases, artificial intelligence, organoid models, human-AI symbiosis

## Abstract

Neurodegenerative diseases, including Alzheimer’s disease (AD) and Parkinson’s disease (PD), present a growing public health challenge globally. Recent advancements in neurotechnology and neuroengineering have significantly enhanced brain–computer interfaces, artificial intelligence, and organoid technologies, making them pivotal instruments for diagnosis, monitoring, disease modeling, treatment development, and rehabilitation of various diseases. Nonetheless, the majority of neural interface platforms focus on unidirectional control paradigms, neglecting the need for co-adaptive systems where both the human user and the interface continually learn and adapt. This selected review consolidates information from neuroscience, artificial intelligence, and organoid engineering to identify the conceptual underpinnings of co-adaptive and symbiotic human–machine interaction. We emphasize significant shortcomings in the advancement of long-term AI-facilitated co-adaptation, which permits individualized diagnostics and progression tracking in Alzheimer’s disease and Parkinson’s disease. We concentrate on incorporating deep learning for adaptive decoding, reinforcement learning for bidirectional feedback, and hybrid organoid–brain–computer interface platforms to mimic disease dynamics and expedite therapy discoveries. This study outlines the trends and limitations of the topics at hand, proposing a research framework for next-generation AI-enhanced neural interfaces targeting neurodegenerative diseases and neurological disorders that are both technologically sophisticated and clinically viable, while adhering to ethical standards.

## 1. Introduction

There are various neurodegenerative diseases (ND), but we focus on Alzheimer’s disease (AD) and Parkinson’s disease (PD), which pose significant challenges for modern neuroscience because they present both major healthcare issues and complex scientific obstacles. These NDs have led to continuous brain deterioration and neuron death, which affects millions of people throughout the world. The Alzheimer’s Association reports that 7 million Americans had Alzheimer’s disease in 2025, but this number will reach 13 million by 2050. Healthcare expenses for Alzheimer’s disease and dementia-related conditions will increase from USD 384 billion in 2025 to nearly USD 1 trillion by 2050, according to current projections [1]. The second most prevalent neurodegenerative disorder after Alzheimer’s disease affects 1 million patients in the United States while generating healthcare expenses of USD 51.9 billion during 2017. The number of Parkinson’s disease patients will surpass 1.6 million while the associated healthcare expenses will reach USD 79 billion by 2037 [2,3]. The rising prevalence of diseases and healthcare expenses requires the immediate development of innovative therapeutic solutions that can surpass current treatment methods. The combination of modern neural interface technology with artificial intelligence (AI) presents a promising solution to develop new treatments for neurodegenerative diseases.

Neuroscience and neuroengineering have established brain–machine interfaces (BMIs) as their fundamental technology, enabling artificial brain–device communication through neuromuscular bypasses. The development of external devices continues with brain–computer interfaces (BCIs), neuroprosthetics, and rehabilitation-assistive technology. The process of BMI operation starts with the recording of neural signals through electrodes or EEG, followed by computational signal processing to extract neurological data, which then controls external devices such as cursors, prosthetic limbs, bots, wheelchairs, and communication tools [4,5]. BMI performance depends on the synchronized activity of multiple areas of the brain, including the motor cortex, the prefrontal cortex, and the striatum, according to research findings [6–10]. The main recurring problem with BMIs is that they require more than a simple installation. The success of BMIs depends on the user’s adaptation to the machine through plasticity, cognitive strategies, and algorithmic learning processes. The evolution of BMI control has progressed beyond basic motor mapping, as researchers now recognize that intention decoding, reinforcement signals, and affective states play a crucial role in the development of adaptive, human-centered systems.

Artificial intelligence (AI) has transformed neurodegenerative disease research through its parallel development. Traditional diagnostic methods based on neuroimaging and linear models have shown limited progress in detecting diseases early and identifying patient subgroups. The field of neurodegenerative disease research has expanded significantly through the implementation of machine learning (ML) and deep learning (DL) techniques. AI-driven pipelines that utilize the multimodal fusion of MRI, PET, and functional imaging data, along with predictive modeling of genetic and proteomic profiles, outperform traditional statistical methods in routine applications [11,12]. The combination of CNNs with RNNs enables researchers to achieve accuracy greater than 90% in AD diagnosis and create precise predictions for the progression of cognitive decline [13,14]. Graph-based methods represent an emerging approach that addresses the non-Euclidean structure of brain connectivity. The application of graph neural networks (GNNs) for brain graph analysis produces better results in AD and MCI classification through their ability to analyze brain network topology [15,16]. The research shows that disease indicators spread throughout interconnected networks, rather than remaining within specific brain areas, which makes holistic modeling essential.

The ability of DL to learn hierarchical spatiotemporal representations from raw data makes it a powerful framework for decoding complex neural signals. The implementation of different architectures for BCIs and neural monitoring systems requires specific adaptations to handle particular modality-related problems. The extraction of localized spatial features from multichannel EEG or fMRI signals through convolutional neural networks (CNNs) becomes possible because their convolutional kernels detect inter-electrode and voxel correlations while preserving temporal stability according to [17]. The sequential EEG and ERP data processing benefits from the use of recurrent neural networks (RNNs) and their gated variants, including long short-term memory (LSTM) and bidirectional LSTM (BiLSTM) for temporal dependency modeling [18]. The combination of CNN and LSTM layers in hybrid architectures allows for simultaneous learning of spatial and temporal patterns, which enhances the detection of P300 and motor imagery signals. The neural decoding field now uses variational autoencoders (VAEs) and transformer-based models to perform tasks that require strong feature compression and extended attention capabilities [19]. The probabilistic nature of VAEs produces noise-free neural representations, which makes them suitable for denoising applications and cross-subject generalization. The transformer encoder uses self-attention to detect distant temporal relationships without needing recurrent connections. The deep architectures become more effective when biomedical priors such as spectral filters and electrode topology and physiological constraints are integrated to enhance both interpretability and brain physiology alignment. The combination of deep learning techniques with neural interface design has created a new generation of BCI systems that adapt to noise and maintain generalizability.

To further strengthen the link between computational neuroscience and biophysical realism, integrate physics-based modeling frameworks into the simulation and analysis of neuroelectronic and organoid systems to enhance the connection between computational neuroscience and biophysical realism. Physics-informed modeling enables researchers to model mechanical deformation and ionic diffusion and heat transfer and electromagnetic field propagation between biological tissue and microelectronic interfaces. The training process of deep learning models benefits from physics-based approaches, which incorporate governing physical laws such as Maxwell’s equations for field dynamics and the bio-heat equation for tissue thermoregulation as structural priors. The recent development of hybrid AI-FEM frameworks shows that deep learning systems can work with physics solvers to perform fast real-time tissue-device interaction simulations at high accuracy levels according to [20]. The paradigm allows for adaptive parameter estimation and fast inverse modeling and better generalization to new physiological states, which are essential for precise neural signal transmission and electrode impedance and bioelectronic temperature rise predictions. The combination of FEM-based stress and diffusion models with AI-driven growth and electrophysiological predictions through organoid studies would create a single framework to study neurodevelopmental processes under electronic stimulation at multiple scales. The integration of biomedical priors into deep learning models through spectral filters and electrode topology and physiological constraints leads to better interpretability and improved brain physiology alignment. The integration of deep learning methods with neural interface development has led to advanced BCI systems that learn from noise while preserving their ability to work across different situations.

The acquisition of electrophysiological signals through electronic front-end architectures continues to be the primary method because these systems deliver high temporal precision and low latency alongside compatibility with MEA and CMOS integration technologies. The systems use low-noise amplifiers (LNAs) and analog-to-digital converters (ADCs) with multiplexing circuits that optimize the recording of microvolt-level potentials at high signal-to-noise ratios (SNRs) across 0.1–10 kHz frequency bands [21]. The increasing number of recording channels and spatial resolution in neural systems creates problems for traditional EFEs because they generate crosstalk and produce thermal noise while consuming excessive power in dense implantable systems. The transduction mechanisms of photonic and SPAD-based (single-photon avalanche diode) systems operate through optical or photon-counting methods to detect neural activity by monitoring calcium or voltage-sensitive fluorescent signals [22]. The nanosecond precision of SPAD arrays makes them suitable for detecting transient neural events because they provide high-sensitivity time-resolved optical detection. The use of photonic interfaces helps reduce electrical artifact contamination and capacitive coupling, which represent major problems in electronic systems. The use of photonic systems requires external fluorescent markers or optogenetic alterations, yet they face restrictions from limited tissue penetration and phototoxicity and thermal damage caused by light stimulation. The direct electrophysiological signal quality of electronic EFEs remains superior to photonic and SPAD-based systems, which provide better non-contact imaging capabilities for large-scale neural dynamic monitoring. The design of high-density neuroelectronic recording systems with low-artifact performance becomes possible through the emerging field of optoelectronic hybrid front-end technology.

The current research has made significant progress, but multiple essential obstacles persist. AI models excel at prediction, but their lack of interpretability prevents scientists from gaining mechanistic understanding and medical professionals from trusting their results. The controlled laboratory success of BMIs for motor restoration often fails to translate to real-world applications due to operational challenges arising from neural fluctuations, patient exhaustion, and device reliability issues. The majority of BMI applications focus on motor function restoration, yet they do not address the cognitive and affective symptoms that define Alzheimer’s disease. The current models, including computational and biological systems, fail to represent the complete dynamic nature of human brain operations. The discovery of organoid biology, combined with AI-powered neural interfaces, has created a new research direction. Scientists use pluripotent stem cells to generate brain organoids, which provide researchers with their first opportunity to study human-specific neurodevelopmental and pathological processes in laboratory settings [23,24]. Organoids duplicate essential features of human brain structure, neural connections, and brain wave patterns that occur during disease states. The combination of advanced neuroelectronic interfaces with spatio-temporal graph neural networks and cyborg organoid platforms enables researchers to conduct closed-loop experiments and perform high-resolution phenotyping and drug screening at unprecedented levels of precision. These systems would allow scientists to study human brain function ethically and mechanistically outside the body, while simultaneously accelerating therapeutic development, according to Wu et al. (2024) [25].

The combination of AI and BCI technology advancements creates new possibilities to address these problems. The development of BCIs now enables people with neurological disabilities to convert their brain signals into functional outputs, allowing them to interact with their surroundings. The development of speech BCIs has reached a high level of accuracy, as researchers have successfully translated brain activity into text or speech synthesis, according to a 2023 study [26]. The company Neuralink has made significant progress in BCI technology through human device implantations, enabling patients to regain their ability to communicate and move. The startup Paradromics has conducted BCI implant tests that convert neural signals into speech and cursor movements to advance clinical usage [27].

Emerging organoid-based neural platforms are providing a novel testbed for advancing BCI technologies [25]. Brain organoids can simulate neurogenesis, neuronal migration, cortical stratification, and neural circuits of the human brain [25,28]. The integration of microelectrode arrays (MEAs) with organoids allows scientists to monitor and control neural activity in real time [25]. The cyborg organoid platform includes the hippocampal cyborg organoid (cyb-organoid) system, which connects human hippocampal organoids (hHOs) to a liquid metal–polymer conductor (MPC)-based mesh neuro-interface [25]. This mesh MPC (mMPC) integrates 128-channel high-density multielectrode arrays that are stretchable (up to 500%) and flexible, facilitating attachment to the hHOs and enabling the detection of neural activity [25]. Furthermore, organoid–brain–computer interfaces (OBCIs) have been developed, representing innovative BCIs mediated by implantable organoids that are capable of bidirectional communication with host tissue [28]. OBCIs aim to repair damaged neural circuits by promoting the regeneration and regulation of neural functions, with the potential to contribute to future bidirectional BCI frameworks implementing feedback and closed-loop regulation [28]. Traditionally, BCIs are characterized by the type of neural activity they record and decode. The established criteria for BCIs include EEG indicators, which include alpha rhythm (8–12 Hz) for relaxed wakefulness and visual evoked potentials (VEPs) and steady-state VEPs (SSVEPs) for periodic visual stimuli and event-related potentials (ERPs), with P300 being the most popular for spelling and selection tasks [4,29]. The use of fMRI, MEG and NIRS enables researchers to develop hybrid BCIs that merge high temporal accuracy with enhanced spatial detail. The fundamental elements of signal selection in BCI research depend on electrophysiological and hemodynamic criteria. The clinical applications of BCIs reach further than AD and PD, although our discussion focused on these two conditions. The practical applications of BCI systems have focused primarily on two neurological conditions, which are amyotrophic lateral sclerosis (ALS) and multiple sclerosis (MS). The combination of P300-based spellers and steady-state visually evoked potential (SSVEP) paradigms enables patients with severe paralysis from ALS to regain communication abilities through brain–computer interface systems [30–32]. The application of BCI systems in MS extends beyond communication to include motor rehabilitation and cognitive neurofeedback, which utilize P300 and ERP-based protocols to boost remaining neural plasticity, according to research by Kuebler et al. (2007) and Guger et al. (2009) [33,34].

The integration of electronic interfaces with biological tissue for safe operation stands as a fundamental challenge in current BCI research. The interface between biological tissue and electronic circuitry through neural interfaces faces two main challenges because electromagnetic energy absorption and heat dissipation affect both device operation and tissue health. The human body requires continuous monitoring of specific absorption rate (SAR) and temperature changes when using devices for extended periods, especially when used indoors or as wearable technology, according to research by [35–37]. The integration of flexible sensors with wireless telemetry in smart electronic systems allows real-time monitoring of SAR and thermal changes, which supports the safe operation of high-density neural recording or stimulation platforms. The integration of biomedical safety frameworks into neural interface research enables the development of next-generation BCIs that combine functional reliability with physiological tolerance.

These advanced systems support the study and modeling of network dynamics relevant to neurodegenerative diseases, such as schizophrenia and Alzheimer’s disease, as hHOs serve as promising models for these conditions [25]. They offer an ethically viable platform for prototyping adaptive neural interfaces [25]. “The technological progress seeks to bring back lost abilities through limb reanimation, memory enhancement, and sensory restoration while creating opportunities to boost human brain capabilities and develop machine–human collaboration systems [38]. The technology provides fresh possibilities for treating patients who have Alzheimer’s disease and Parkinson’s disease [38].

The literature shows substantial progress in BCIs and Artificial Intelligence (AI), yet researchers have not thoroughly investigated how humans and machines adapt to each other during interactions [38]. The current BCI systems focus on one-way adaptation because users learn to operate the system by decoding their neural signals to control external devices [38,39]. The scientific community has not thoroughly studied mutual adaptation between humans and AI systems, which involves their joint development for performance improvement [38,40]. Human–machine interface operation functions as a “two-learners problem,” yet researchers have conducted minimal studies about the specific processes through which users develop effective control methods for interface interaction [39,40]. Brain co-processors, for example, are designed for joint optimization of cost functions with the nervous system, signifying a move towards mutual adaptation [38].

Building on these gaps, a recent analysis by Yang (2024) identifies additional technical, ethical, and societal limitations in applying BCIs to AD. The first difficulty arises from signal quality, as external interference, natural bodily changes, and emotional responses easily influence cognitive state-related brain signals. The process of obtaining reliable neural information for cognitive rehabilitation programs proves to be extremely difficult. The use of extensive or invasive BCI devices creates patient discomfort, which negatively impacts their ability to follow treatment and maintain system usage in the long term [41]. The process of obtaining brain data raises ethical concerns due to its involvement in handling sensitive and private information. The mental decline of AD patients prevents them from giving consent and safeguarding their rights because they cannot make complete decisions. The high cost of BCI systems creates social problems because it limits their accessibility to specific groups of people, thereby intensifying existing healthcare inequalities. The combination of BCIs with AI, big data, and portable hardware technology will lead to improved Alzheimer’s disease (AD) diagnosis, more precise interventions, and enhanced preventive monitoring capabilities. The research community has not studied the co-adaptive learning process between patients and systems, which requires extensive mutual learning that spans months to years. Researchers must address critical knowledge gaps in developing BCIs for neurodegenerative disease stages to create systems that integrate technical excellence, ethical standards, human focus, and clinical effectiveness.

The primary objective of this selective thematic review is to explore and synthesize existing literature on neural interfaces, organoid models, and AI, with a focus on key themes such as AI and ML, neural interfaces and brain organoids, and human–AI symbiosis. This review offers insights into research gaps, the evolving understanding, and the practical relevance. It organizes existing evidence around the development of co-adaptive brain–computer interfaces for individuals living with neurodegenerative diseases, specifically AD and PD. These conditions are marked by progressive deterioration of motor, cognitive, and/or communicative abilities, necessitating assistive technologies that are responsive and dynamically adaptable to the user’s evolving neurological state. Traditional BCIs have focused on signal decoding and external control, without sufficient attention to co-adaptation, the mutual learning and adjustment between human users and artificial systems. This paper addresses that gap by proposing a scoping review of technologies and frameworks that enable human–AI symbiosis in assistive and therapeutic applications for AD/PD populations. The specific objectives of this review article are as follows:

### 1. Map Key Concept of Co-adaptive Symbiotic Interactions

Co-adaptive neural interface systems represent a progression beyond traditional BCIs by facilitating mutual learning between human users and AI-controlled systems. The systems operate through a two-way process (bi-directional) that enables both user neural signals and interface decoding algorithms to adapt simultaneously. The systems require this capability because neurodegenerative diseases like AD and PD cause users to lose their ability to express intentions while their motor and cognitive functions deteriorate. We trace the development of neural interface technologies and their integration with brain organoids, high-density MEAs, and deep learning models. Key architectural components include the following:

- Signal acquisition modules (e.g., EEG, ECoG, intracortical probes).
- Real-time pattern recognition occurs through recurrent neural networks (e.g., BiLSTM, GRU) for pattern recognition.
- The system utilizes adaptive feedback loops that implement reinforcement learning or unsupervised adaptation methods.
- Embodied interaction models, as seen in passive BCIs decoding multi-dimensional mental states (MDMS), e.g., stress, engagement.

Human–AI symbiosis, as described here, operates through co-regulation and personalization, as AI receives neural signal data from users, while users modify their intent strategies based on system feedback. The system maintains its ability to adapt through continuous operation, which proves essential for treating patients with long-term neurodegenerative diseases.

### 2. Identifying Gaps in the Literature on Co-Adaptive BCI for AD/PD

The application of BCI technology to neurodegenerative patient populations faces multiple challenges for the following reasons:

- Limited personalization over disease progression: The current BCI systems maintain fixed calibration methods, which fail to support the time-dependent cognitive and motor deterioration patterns found in AD and PD patients.
- The majority of BCI systems operate as one-way control systems, but researchers have proven that feedback-driven models work effectively in non-clinical environments through studies like DishBrain and Dehais et al.’s flight simulation BCI.
- Sparse integration of organoids in real-time systems: Brain organoids remain largely exploratory and preclinical, and their potential as adaptive computational substrates in BCI feedback loops has not been fully realized.
- Lack of neurophenomenological decoding: Few systems address the first-person experience, which is critical for assessing intention, emotional valence, and motivational state factors, especially relevant in disorders with affective-cognitive comorbidity.
- Insufficient long-term tracking infrastructure: There is a lack of frameworks to track patient-specific neural trajectories and tailor interface adaptation accordingly over months or years.

### 3. Synthesizing Evidence Across Neuroscience, AI, and Clinical Studies

Drawing from the reviewed literature, we synthesize a unified framework for next-generation BCIs in neurodegenerative contexts. Bidirectional BCIs that integrate deep learning, neural feedback, and biologically relevant models (e.g., organoids or personalized MEG/EEG profiles) offer the following advantages:

- Enhanced communication: Adaptive BCIs can restore interaction capacity in late-stage AD or advanced PD patients by mapping residual neural intent to assistive outputs.
- Improved mobility and motor planning: The system achieves better motor control and planning through real-time co-adaptation with recurrent models (LSTM, BiLSTM), which produces stable intent decoding during neural system changes.
- Personalized treatment response tracking: AI systems that analyze MEG/fMRI or electrophysiological changes in organoid models of PD midbrain can predict and track the effectiveness of pharmacologic or DBS treatments.
- The integration of neurophenomenological data through passive BCIs enables interfaces to detect user state changes beyond basic command input for creating cognitive-affective interfaces.
- Closed-loop hybrid systems: Emerging platforms, such as organoid–AI symbiosis, create the possibility for feedback-conditioned drug screening, personalized to a patient’s neural phenotype and integrated into BCI control pipelines.

### 4. Thematic Focus and Methodology

This thematic review aims to explore and synthesize emerging literature on co-adaptive neural interfaces and brain organoid integration for neurodegenerative disorders, with a particular focus on the following:

1. Adaptive AI architectures in brain–computer interfaces.
2. Organoid-based neuroengineering platforms for modeling and intervention.
3. Bidirectional and personalized feedback loops for AD and PD patients.

We seek to provide a conceptual and translational framework for developing next-generation BCIs that evolve with the user, offering sustainable, ethically sound strategies for managing cognitive and motor decline. This thematic review contributes a conceptual and translational framework for developing co-adaptive neural interface technologies tailored to neurodegenerative disorders. By mapping foundational concepts of symbiotic human–AI interaction, identifying implementation gaps in BCI design for AD/PD, and synthesizing interdisciplinary evidence from neural engineering and clinical neuroscience, we propose a path forward for sustainable, ethically informed, and biologically integrated BCI systems. In particular, the convergence of deep learning, brain organoids, and co-adaptive feedback architectures presents new possibilities for restoring communication, enhancing mobility, and improving cognitive function in individuals affected by these conditions. Ultimately, human–AI symbiosis in this context represents not only a technological innovation but also a paradigm shift toward empathetic and personalized neurotechnological care.

## 2. AI and Machine Learning in Neuroscience and Neuroengineering

Neuroscience is dedicated to the study of the nervous system. It explores multiple aspects of the brain and nervous system, such as their structure, function, development, and the impact of diseases on them [42]. Neuroscience is a scientific field that studies the nervous system. It explores multiple aspects of the brain and nervous system, including their physical organization and operational mechanisms, as well as their developmental processes and disease-related effects [42]. Neuroengineering is a multidisciplinary discipline that connects neuroscience with engineering, computer science, and biomedical sciences. The primary objective of neuroengineering involves studying neural systems through the development of devices and computational tools to diagnose, restore, or enhance neural functions [43]. Neuroengineering emerged as an independent field during the late 20th century, driven by the expansion of electrophysiology and biomedical engineering research. The cochlear implant invention, along with the development of deep brain stimulation (DBS), has established significant breakthroughs in neuroengineering [44].

AI and ML systems are transforming neuroscience and neuroengineering by enabling the analysis of extensive and complex neural data at scale, thereby creating adaptive brain–machine interfaces. These technologies help scientists understand brain operations while creating neural models and enabling medical treatments. AI systems analyze extensive datasets that combine electrophysiological recordings with imaging results and behavioral measurements. The combination of DL methods, including CNNs, RNNs, and VAEs, enables better spike train analysis and calcium imaging. At the same time, VAEs serve as effective encoding models to reveal neural representation patterns [45]. DL models outperform conventional classifiers when analyzing multimodal imaging data that includes MRI and PET to detect neurodegenerative and psychiatric conditions in neurodiagnostics. Deep learning architectures demonstrate exceptional performance in diagnosing Alzheimer’s disease, Parkinson’s disease, and schizophrenia, thanks to their high diagnostic precision. The application of transfer learning improves model performance when working with restricted datasets that contain limited labeled information [46]. AI models serve as the foundation for neuroengineering to create brain–computer interfaces, which aim to enhance or restore the operations of the neural system. The application of DL methods to EEG, ECoG and intracortical recordings enables users to control prosthetic devices through intention detection and restore communication abilities for paralyzed individuals. Real-time decoding operations benefit from recurrent models, which include LSTM networks, because they provide stable performance [47].

The integration of AI and ML technology has significantly advanced neuroengineering by enabling sophisticated neural data analysis, developing brain–computer interfaces (BCIs), and creating adaptive neuroprosthetics. AI has revolutionized neuroengineering by allowing the discovery of new brain mechanisms and enhancing our understanding of brain function and disorders. AI analysis of brain dysfunction has generated substantial knowledge about neurological disorders, which in turn improves diagnostic methods and treatment approaches and enhances traditional clinical procedures. ML and AI methods are applied in neuroengineering to study brain signals and create brain–computer interfaces and improve neurological disorder treatments. AI and ML enable scientists to read brain signals and predict neurological events, creating individualized treatments that open new possibilities for brain research and human performance enhancement [48]. The field of NeuroAI represents a new trend that unites neuroscience with AI through biological neural process-inspired AI systems. Establishing effective interdisciplinary collaboration in this field requires structured integration of expertise across computational, biological, and clinical domains. A practical method to establish effective interdisciplinary collaboration involves building translational research environments that unite data scientists and engineers with neuroscientists through experimental workflows that connect algorithm development to neural recording and stimulation studies. The exchange of models and experimental results becomes possible through joint data standards (Neurodata Without Borders), co-supervised projects and open neural simulation platforms. The development of professionals who link these fields becomes possible through dual training programs that combine computational neuroscience with bioengineering education. The development of structured connections between fields enables the creation of a systematic approach that speeds up AI-driven neuroengineering research [49]. The concept of the “embodied Turing test” has been proposed. The evaluation of AI models depends on their ability to mimic animal sensorimotor behaviors, which drives AI development toward more adaptable and transferable systems [50,51]. The degree to which AI research adopts neuroscience principles remains a subject of ongoing debate among professionals. The extent to which AI research incorporates neuroscience principles remains a topic of professional debate. One of the ongoing topics of discussion among experts is the extent to which AI research is inspired by neurobiology. Researchers have shifted their focus from biological models, which could have provided critical insights into neural systems, to scaling transformer architecture. As highlighted in Table 1, the incorporation of AI and ML methodologies has greatly enhanced research efforts in Alzheimer’s and Parkinson’s diseases.

The processing of information through brain-inspired computing technologies offers a fundamentally different paradigm for information processing, characterized by high energy efficiency and a superior ability to handle large volumes of unstructured and noisy data from daily life. The achievement of this potential requires that researchers unite their efforts, receiving financial backing and structural resources to advance brain-inspired computing research [73]. Artificial intelligence serves as a transformative power in organoid research, enabling rapid evaluation of construction strategies, efficient analysis of multi-omics data, and precise assessment of preclinical models. The AI-powered organoids would allow scientists to study organ development and disease mechanisms more effectively, creating opportunities for future medical applications [74]. The development of DeepDendrite represents a new GPU-accelerated computing framework that merges dendritic hierarchical scheduling with the NEURON simulator’s GPU engine to support detailed neural simulations. The frameworks enable faster neuroscience research and demonstrate potential methods to merge realistic biological models into standard AI operations for image classification training [75].

## 3. Neural Interfaces and Brain Organoids in Neuroengineering

Neuroengineering research now emphasizes the combination of neural interfaces with human brain organoids as essential tools for studying and treating brain disorders. The direct link between neural circuits and external devices becomes possible through neural interfaces, which include brain–computer interfaces (BCIs) and brain–machine interfaces (BMIs). The technology has proven essential for various applications, including motor rehabilitation and speech decoding. Brain organoids function as three-dimensional, self-organizing human pluripotent stem cell-derived structures that create scalable laboratory models for studying human brain development, connectivity, and disease processes [23,74,76–78]. The combination of these platforms with AI technology, as well as top-tier neuroscience journals *Nature Neuroscience, Neuron, and Nature Methods*, enables researchers to develop new experimental methods for studying neural patterns, disease modeling, and therapeutic development for Alzheimer’s and Parkinson’s diseases [79–81]. The National Institutes of Health (NIH) identifies organoid systems combined with AI-driven neural interfaces as a groundbreaking advancement in brain science, which supports the BRAIN Initiative’s goals for developing scalable, multimodal model systems [50,51,82].

Brain organoids are three-dimensional neural tissues that are derived from human pluripotent stem cells that develop into layered structures containing progenitors and neurons and glia cells, which replicate the initial stages of human cortical development [83–85]. In contrast to traditional two-dimensional cultures, they reproduce not only cellular diversity but also tissue-level processes such as neurogenesis, neuronal migration, cortical stratification, and early circuit formation [76,86]. The organoids demonstrate their ability to join existing neural networks when scientists place them into sensory, motor, or visual cortex areas of host animals because they survive and develop while sending their axons throughout the surrounding neural tissue [87–90]. The transplantation of immature organoids containing numerous ventricular-zone progenitors leads to uncontrolled cell division; however, their random neural projections prevent the formation of precise host–graft neural connections. In vivo experiments that combine electrical and optogenetic neural stimulation lead to faster organoid development, directed neuronal cell growth and migration, and target-oriented axonal extension, resulting in improved structural and functional integration as well as reduced progenitor cell proliferation [76,91].

### 3.1. In Vivo Neural Interfaces

Neural interfaces are bioelectronic systems that establish direct communication links between nervous system functions and digital technology platforms [92]. The technology behind neural interfaces has evolved from its original use in the human brain, spinal cord, and peripheral nerves to create new hybrid biological-artificial intelligence systems, including brain organoids. The development of BCIs and BMIs has led to groundbreaking applications, which include ECoG signal-based speech decoding and prosthetic and virtual device control [26,93,94]. Neuralink represents a notable example of an implantable BCI system that connects directly to cortical neurons to enable motor and communication restoration [95]. The technologies convert brain signals into functional commands, enhancing the independence, communication abilities, and mobility of people with severe motor disabilities [96–98]. The current non-invasive systems face ongoing challenges because they operate at limited bandwidth and produce low signal-to-noise ratios [99,100].

The human brain, comprising 86 billion neurons and trillions of synaptic connections, has been the primary focus of neuroscience and BCI research since its discovery [101]. The study of the human brain faces multiple obstacles, such as inaccessibility, ethical restrictions, and the challenge of monitoring cellular activities in vivo [102]. The development of brain organoids became necessary because researchers needed to study human neural functions in laboratory-controlled systems.

The long-term sustainability of neural interface technologies faces ongoing obstacles that prevent their complete resolution. The primary challenge of biocompatibility arises from chronic immune responses and foreign-body reactions, which lead to tissue damage and deterioration of signal quality near the implant site [103]. The combination of rigid electrode materials with neural tissue structures exacerbates inflammation and gliosis during extended implantation periods due to their mechanical incompatibility [104,105]. High-density electrode arrays provide better spatial resolution, but their recordings become less accurate because of electrical crosstalk that occurs when fields overlap [106]. The devices experience degradation through electrochemical corrosion, mechanical strain, and material fatigue, which reduces their operational stability and medical trustworthiness over time [107].

### 3.2. In Vitro Brain Organoid Platforms

Brain organoids are three-dimensional neural tissues that contain diverse cell types, including neurons and glia, closely mimicking those in the human brain [74,78,102]. The three-dimensional structure of organoids surpasses traditional 2D cultures and animal models by creating brain structures that accurately represent human brain development and disease progression [23,108]. The use of patient-derived models enables researchers to study disease mechanisms at various developmental stages, thereby advancing precision medical approaches. Scientists use high-resolution microelectrode array (MEA) systems from MaxWell Biosystems to record spontaneous neural activity and functional brain connections and oscillations in real-time [76,79].

One of the most compelling demonstrations of organoid-based neuroadaptive behavior is the DishBrain project, in which researchers trained living neurons cultured in vitro to play a simplified version of the game Pong. The 2022 research by Kagan et al. demonstrated a closed-loop system that used feedback learning to show that neuronal networks could develop goal-oriented behavior through environmental stimulus responses [109]. The operational sequence followed a well-defined loop consisting of signal acquisition → feature extraction → state prediction → feedback evaluation → electrical stimulation → network adaptation, aligning with predictive-coding principles proposed for adaptive computation in biological networks. The study illustrates how organoids can function as intelligent agents in AI-integrated platforms, as it demonstrates successful real-time communication between biological and digital systems.

In the context of BMI, organoids enable researchers to build closed-loop hybrid systems for BMI applications through AI-driven neural interfaces that interpret organoid activity in real time [80]. The new research approach enables scientists to study neural computation that emerges from brain activity, rather than trying to decode human intentions. Deep learning models show their ability to interpret intricate patterns in electrophysiological signals. The research by Trujillo et al. (2019) showed that organoid electrical signals progress toward mature oscillatory patterns, which unsupervised learning models can effectively predict [77]. The research field uses convolutional neural networks and spatiotemporal graph neural networks to extract neural network connections and dynamic patterns from biological data [110].

**Figure 1.**
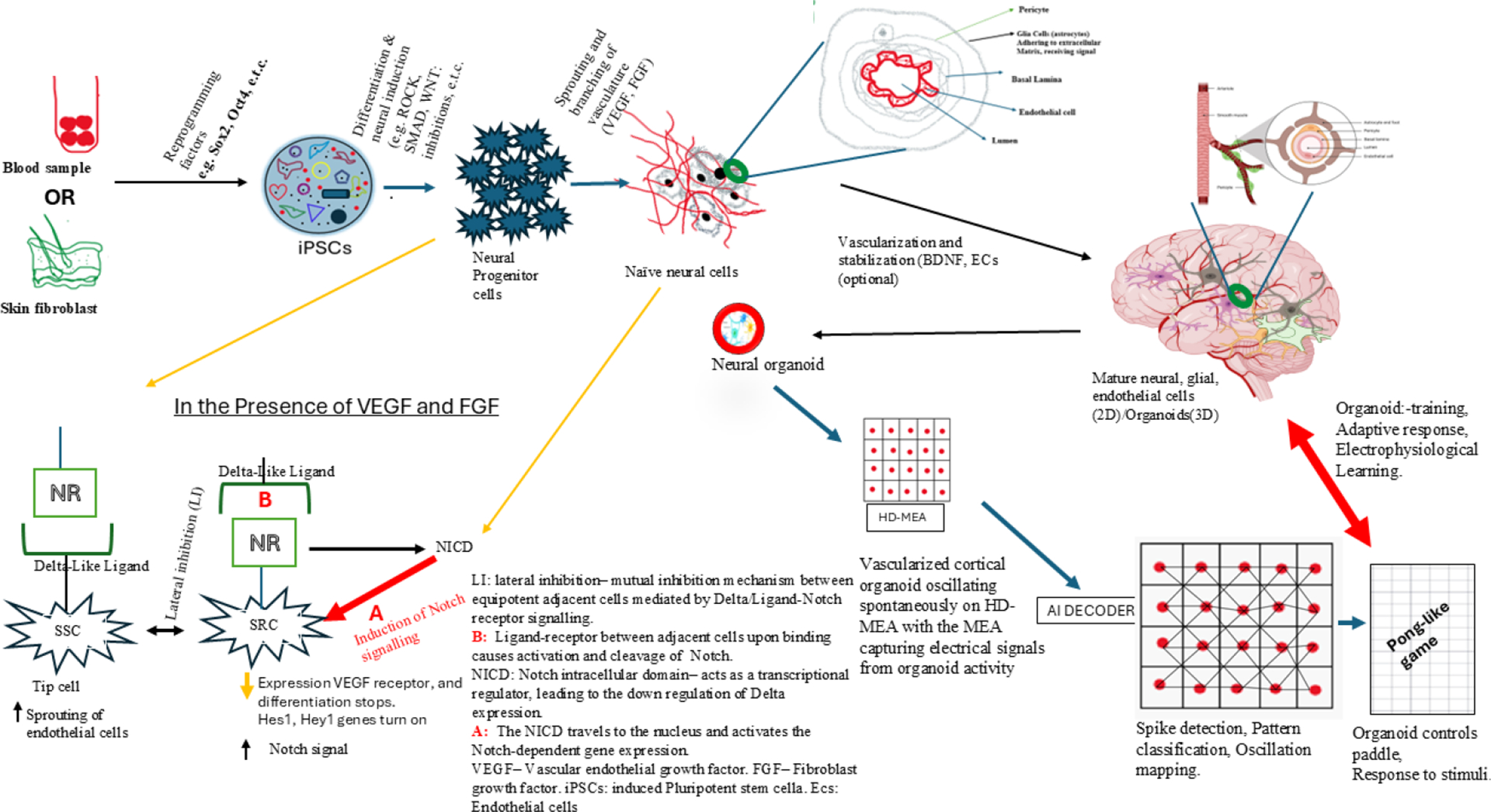
In vitro differentiation, vascularization, and machine interface of neural organoid [117,118,124,136].

By integrating organoids and neural interfaces, researchers can create ethically acceptable neural systems, resulting in reduced invasiveness. The systems offer dense, high-resolution recording capabilities that human brains lack due to ethical and technological limitations. The systems prove best for studying early disease progression, drug testing, and neurotechnology development. The complete utilization of brain organoids in these platforms depends on achieving long-term viability and functional maturation, which requires vascularization strategies to support their growth. Figure 1 provides a schematic overview of in-vitro vascularized organoid development and integration with microelectrode array (MEA) systems. The illustration summarizes how human induced pluripotent stem cells (iPSCs), derived from skin fibroblasts or blood cells, differentiate into neural progenitor cells and subsequently form neural organoids under the influence of growth factors such as VEGF and FGF. Within these systems, Notch signaling governs endothelial sprouting and vascular network formation, leading to the development of perfusable cortical organoids capable of electrophysiological learning and closed-loop AI interaction (adapted from this study’s conceptual model). The addition of in vitro vascularization systems provides better oxygen and nutrient supply to cells while preventing necrotic core development and enabling the modeling of neurovascular interactions. The combination of EC co-culture with co-differentiation and assembloids, as well as organ-on-chip systems and engineered ECM, has proven effective for cortical organoid development. The integration of perfusable vascular networks into MEA-compatible organoid cultures produces better electrophysiological stability and more accurate functional measurements [111–115]. These advancements collectively position vascularized cortical organoids as revolutionary instruments for neuroengineering research. The combination of deep learning systems with these biological systems enables researchers to conduct closed-loop experiments, train models, and achieve co-adaptive learning between biological systems and artificial intelligence. The developed technologies pave the way for advanced neural interfaces, which will establish a new brain–machine partnership beyond current BMI systems.

Human iPSCs aggregate to form early organoids that are capable of differentiating into various cell types in low-adhesion plates. In the presence of ROCK, SMAD inhibitors, and other factors, differentiated neural progenitor cells located near the core give rise to neurons/glial cells. SSC (signal-sending cells), which possess healthy tip cells that express a ligand, activate Notch signaling in neighboring cells. This step reinforces their identity as a tip cell, as they sense gradients of VEGF produced by the organoid’s neural progenitors or hypoxic core.

SRC (signal-receiving cells) receives and responds to the notch signal (forms the endothelial stalk cells). This Notch activation suppresses their VEGF receptor expression, halting their differentiation while allowing the tip cells to sprout. The process of Notch signaling facilitates the formation of capillary-like networks of infiltrating endothelial cells (ECs) that organize into vessels within and around the organoid, enabling active nutrient/oxygen transportation. The ECs can be added to the organoids or embedded in the ECM, depending on the culture vessel in use (Refs. [116–137]).

### 3.3. Integrated Summary of Neural Interface Systems and Organoid Models in AD and PD

Interactions that had been found to play essential roles in BMI control include cortico-striatal plasticity for motor control and cortico-striatal and cortico-cortical for abstract skill learning, the cortico-striatal thalamo-cortical feedback loop for motor control, and the cortico-basal ganglia–thalamo-cortical loop for understanding cognition, behavior, and the nature of neurological disorders [138–141].

Several complex neurological disorders arise due to alterations in specific or combined malfunctional circuitry of subnetworks of interactions [142,143]; for instance Parkinson’s disease in humans is typically due to decreased activity of the globus pallidus external (GPe) part of the neurons [144,145]; seizures and epilepsy manifest in intracellular Cl-dysregulation [146]. AD, characterized by amyloid (A*β*) plaques and Tau misfolded and hyperphosphorylated protein tangles, has been suggested by Grieco et al. (2023) [147] that neural activity dysregulation may lead to abnormal neural circuit function. In Parkinson’s disease, research has shown that the potential pathology of alpha-synuclein and neural activity is mediated through changes in the dynamics of synaptic vesicles [148]. This relationship is bidirectional [149].

The benefits of brain organoids cannot be overemphasized, especially in public health and neurological diseases that are silently becoming a pandemic. In addition to the estimated impact of Alzheimer’s disease on elderly people, one in every six children born in the United States of America alone has a form of neurological disorder [150]; hence, the need for crucial tools like brain organoids that would enable proper understanding of neurological disorders, together with models that facilitate learning and remembering, for appropriate therapeutics to be developed. The changes in neurotransmitter release and synaptic structures have been the beneficial mechanisms of brain stimulation for treating some neurological symptoms and improving patients’ cognition [151–155]. However, measuring neural activity in the induced intracellular changes may help diagnose, monitor progression, and assess treatment strategies for neurological diseases/disorders.

Tables 2–4 provide a consolidated overview of the principal technologies currently used in AD and PD research.

The classification system in Table 2 categorizes neural interface systems according to their invasiveness level, ranging from non-invasive EEG and fNIRS to invasive deep brain stimulation (DBS), while highlighting their benefits, drawbacks, and ethical implications. The classification system in Table 3 categorizes similar technologies into signal-based categories, including electrical, optical, magnetic, and ultrasonic signals, to facilitate readers’ evaluation through modality-specific perspectives. The dual-view framework facilitates a deeper understanding of the neural interface selection and evaluation processes in both clinical and experimental environments.

The research on AD/PD utilizes state-of-the-art organoid models, which include cerebral and midbrain organoids, as well as vascularized and fused patient-derived organoids, as shown in Table 4. The models replicate human neurodevelopmental processes and pathological features, enabling scientists to utilize them in the development of AI-based bioelectronic systems for drug testing, disease simulation, and personalized medical treatments. The tables illustrate how neural engineering, organoid biotechnology, and translational neuroscience are converging to create a unified framework for innovative research in neurodegenerative diseases.

### 3.4. BCI Signal Flow and System Architecture

**Figure 2.**
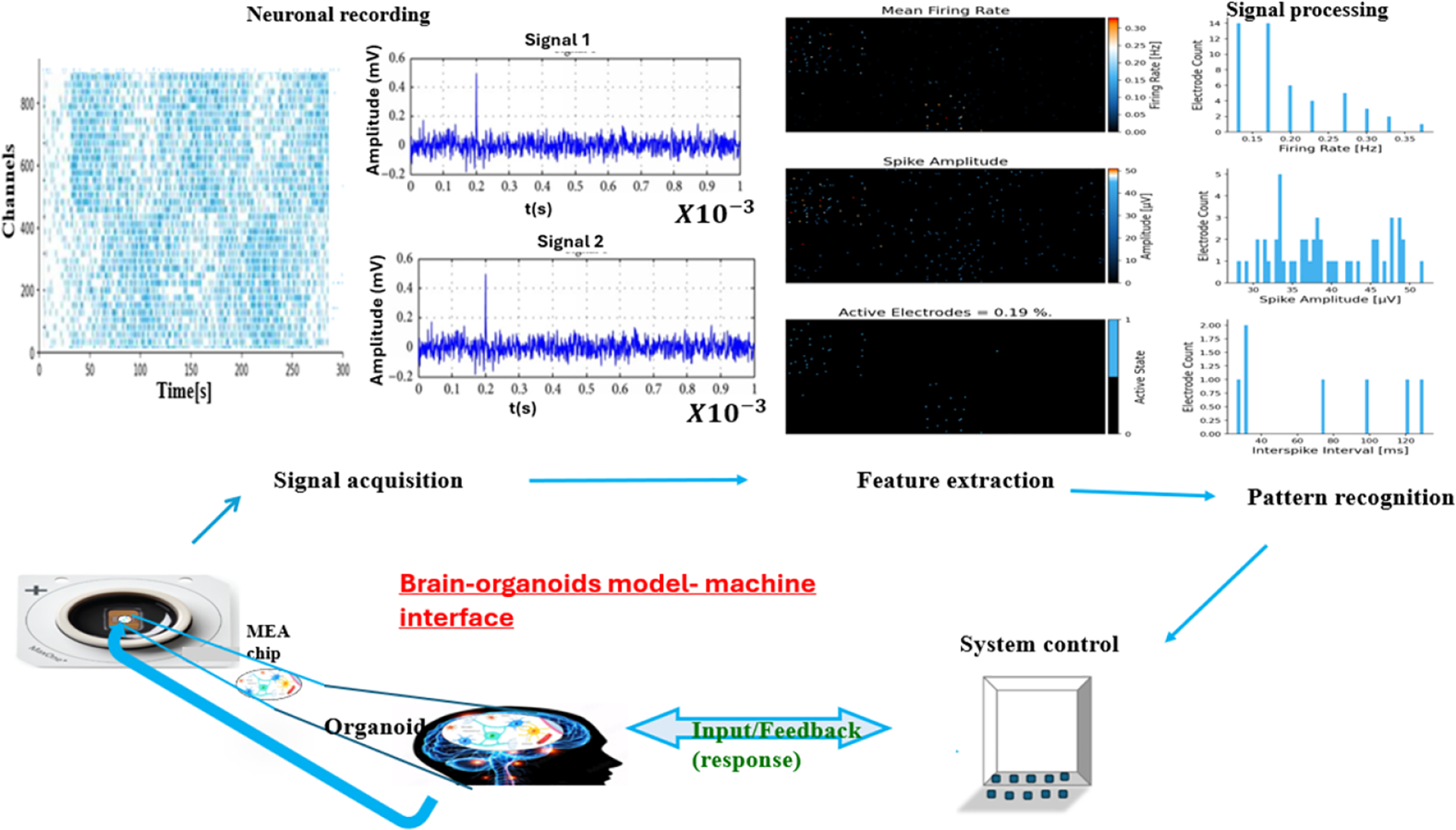
Schematic illustration of brain–organoid model–machine interface.

The typical structure of BCI systems comprises four main stages, beginning with signal acquisition, followed by signal processing that enables feature extraction and pattern recognition, and then control execution, and finally, feedback. The workflow of this process appears in Figure 2. The acquisition of signals occurs through various types of electrodes, including both invasive and non-invasive devices that measure neural activity using EEG, ECoG, and intracortical spikes. This raw data undergoes preprocessing before feature extraction algorithms detect specific neural patterns, which include motor imagery, attention, and speech intention. Real-time control signal generation is achieved through pattern recognition modules, which utilize machine learning and deep learning models to classify extracted features. The system transmits these signals to robotic limbs, communication interfaces, and assistive platforms. The user receives feedback about their intended outcome through a feedback system, which completes the closed-loop interaction. The system design serves as a fundamental requirement for real-time neurorehabilitation and assistive communication systems, which benefit patients who have neurodegenerative diseases, including AD and PD.

## 4. Human–AI Symbiosis: Co-Adaptation and Emerging Paradigms

Human–AI symbiosis is an emerging paradigm in which humans and artificial intelligence systems co-adapt in real-time, creating a dynamic partnership that strengthens their combined abilities. The framework differs from traditional human-computer interaction because it focuses on adaptive processes that integrate cognitive processing with emotional, motivational, and physiological systems.

The co-adaptive BCIs enable users and systems to evolve together through their shared learning experiences. The systems surpass traditional decoding models (unidirectional) to create feedback mechanisms that allow AI to learn from brain signals while assisting users in refining their mental approach. The dual-agent co-adaptation framework developed by De Santis et al. (2023) [40] enables users and interfaces to adapt simultaneously through unsupervised and reinforcement-based mechanisms that operate independently of predetermined task objectives. The interface adjusts its operations through an unsupervised learning method, which optimizes the process of converting user actions into interface responses. Users experience use-dependent adaptation through reinforcement learning, which receives feedback from intrinsic processes during extended periods of use, rather than task-related errors. The system optimizes interaction efficiency through co-adaptation, enabling flexible and robust human–machine collaboration. Neuroadaptive modeling represents a fundamental requirement for this development. The term “neuroadaptive technology” emerged from Zander Labs to describe passive BCIs, which extract MDMS data to train adaptive AI models, as defined by [213]. The system records stress levels and engagement and motivation states from users without needing any direct commands. The system enables machines to understand context and intent through implicit interpretation, which results in more natural user interfaces.

The current traditional BCI methods often fail to detect the personal aspects of perception and intention that people experience directly. The development of future systems requires them to surpass mechanical decoding methods to understand the emotional and subjective aspects of users’ mental processes. Neurophenomenological decoding establishes connections between EEG patterns and conscious experiences such as frustration, insight, and the desire to create systems that understand the motivations behind user actions. The system establishes a complete connection between neural signals and psychological meaning through this loop, enabling actions based on user intent and providing feedback that aligns with their emotional state [214]. The way users perceive AI feedback depends on whether it aligns with their self-image, as this creates a “quasi-self” experience; however, mismatched feedback results in a “quasi-other” perception. The discovery highlights the importance of explainable AI systems, effective interface design, and trust calibration mechanisms in the development of neuroadaptive technology. Zander Labs achieved scalability in personalization through their universal classifiers, which process thousands of datasets, raising concerns about privacy, identity, and autonomy. The system’s ability to detect and forecast emotional states creates fresh moral dilemmas about who should determine the interpretation process. Machines possess the capability to make false representations of human emotions. The process of developing adaptive feedback systems requires methods to protect user autonomy from degradation. The achievement of human–AI symbiosis requires more than technical solutions, as it presents a philosophical dilemma that necessitates designers working together to develop transparent and ethical systems.

An emerging area within human–AI symbiosis is the development of co-adaptive systems, which unite AI technology with brain organoids. AI technology enhances organoid-based disease models and drug screening platforms through a feedback loop, enabling biological substrates to modify computational models. The Brain and Organoid Manifold Alignment (BOMA) system utilizes AI to match gene expression patterns between developing human brains and organoids, thereby enabling advanced research in developmental biology. The combination of biobank data with machine learning algorithms enables the detection of patterns in multi-omics and imaging databases, thereby minimizing human errors and accelerating clinical research operations. The AI–organoid system uses adaptive methods to forecast how drugs will affect patients and their potential toxic side effects. The combination of machine learning algorithms with 3D pharmacogenomic data enables predictions of individual treatment responses, while AI-guided image segmentation tools evaluate vascular differences in tumor organoids. The system allows continuous feedback operations, where organoids adapt to experimental changes and AI models generate updated predictions, leading to new therapeutic approaches. AI systems that study midbrain organoids for the progression of Parkinson’s and Alzheimer’s diseases enable the real-time adaptation of treatment strategies. These platforms demonstrate mutual shaping because organoids transform experimental and AI-directed procedures, while AI models refine their models through biological feedback. The combined approach of preclinical workflow acceleration and personalized therapeutic optimization emerges from this co-adaptive system. The triadic model of human–AI–organoid symbiosis presents itself as a strong framework for future precision neuroscience applications. The Zander Labs team proved that BCI systems that monitor medial prefrontal cortex activity passively enable users to control cursors implicitly, which serves as a basis for AI systems that adapt to human mental states in real-time [213,215]. Dehais et al. created a dual passive–reactive BCI system for flight simulator operations, which combined stress detection with reactive control to execute collision prevention and basic neuroadaptive copilot functionality [216]. The AI systems in these examples utilize pilot emotional and attentional data to make proactive adjustments, thereby decreasing workload while enhancing safety in complex operational settings. The current neuroadaptive frameworks show promise for future development in creative and educational applications because they can detect human emotional and cognitive states to provide real-time content recommendations, interaction tools, and pacing adjustments. Human–AI collaboration has progressed from assistive prosthetic development into domain-independent copilot and co-creative systems, which use neuroadaptive intelligence to support human cognition.

## 5. Challenges and Future Directions

As the fields of neural interfaces and organoid technologies converge with artificial intelligence (AI) and machine learning (ML) to create innovative solutions for studying and managing neurodegenerative diseases, including Alzheimer’s disease (AD) and Parkinson’s disease (PD). The complete realization of these innovations depends on solving current technical obstacles while handling ethical issues that researchers will encounter in their next phase of work.

### 5.1. Challenges and Limitations

Organoid-based neuroengineering development faces multiple significant challenges that impact ethical considerations, experimental procedures, and technological capabilities. Ethically, the growing biological complexity of organoids, especially when combined with artificial intelligence, raises serious concerns around informed consent, sourcing of human biological materials, data privacy, and the ambiguous moral status of advanced organoids that may display signs of sentience or consciousness [217–220]. Technologically, organoids have proven invaluable for modeling human development and disease, yet several limitations constrain their utility. Reproducibility remains a significant concern due to batch variability, manual handling, and dependence on poorly defined matrices, such as Matrigel [221]. Organoids currently exist as fetal tissue models, which do not contain the necessary components for adult physiological modeling because they lack maturation, vascularization, innervation, and stromal elements [24,222]. The current brain organoid systems demonstrate excellent capabilities for mimicking human developmental stages and disease symptoms, yet they are not ready for medical applications. The main applications of these systems exist in drug screening and modeling because their lack of reproducibility and maturation problems makes them unsuitable for therapeutic tissue use. The lack of standardized differentiation methods together with poor vascularization and unstable electrophysiological activity makes it impossible to use these constructs for transplantation or regenerative medicine. The development of standardized ethical and regulatory frameworks for human-derived neural constructs remains ongoing, which creates challenges for their future clinical implementation. The transition from experimental organoids to clinically applicable neurotherapeutic tools needs advancements in long-term vascularized culture methods and automated production systems and standardized quality-control systems that follow Good Manufacturing Practice (GMP) guidelines [24,217,222]. Research on systemic diseases and immune system interactions is limited due to these limitations. The combination of genetic instability and epigenetic changes in extended cell cultures, along with the absence of standard operating procedures for organoid derivation, maintenance, and validation, creates obstacles for large-scale and reliable research [223–226]. The development of fundamental consciousness in brain organoids creates significant ethical problems during research activities [227,228]. Organoid production requires extensive manual work, which prevents researchers from performing large-scale experiments. Their poor vascularization and immune system incompatibility also make them unsuitable for medical applications [229]. The complete utilization of organoid systems requires scientists to develop new methods and technologies that solve multiple existing problems.

The National Institutes of Health (NIH) created the Standardized Organoid Modeling (SOM) Center as an institutional strategy to handle ethical and reproducibility issues during their 25 September 2025 announcement. The SOM Center represents the first national program that creates standardized organoid models that combine reproducibility with ethical oversight and artificial intelligence and robotic enhancement. The center receives USD 87 million during its first three years to establish standardized organoid models that decrease animal testing needs and ensure transparent research practices and ethical compliance. The initiative uses AI optimization and automated production systems and open-access data and protocol repositories to speed up the development of reproducible organoid science and gain regulatory approval. The NIH SOM Center demonstrates how scientific organizations can handle organoid and AI convergence ethical problems through structured oversight and open science and global collaboration, according to [230].

In vivo experiments remain indispensable for validating in vitro findings, yet they introduce significant obstacles. High-quality, artifact-free neural recordings over extended durations and across heterogeneous subject groups are rare; even state-of-the-art transparent neural probes in Nature and Microsystems & Nanoengineering face persistent challenges in minimizing electrical and optical artifacts during live neural recording [231–233]. Without detailed solutions, translation into clinical or ecological validity will remain constrained. Lastly, the rapid proliferation of sophisticated recording and stimulation technologies has outpaced existing computational infrastructure. The data generated from dense, high-throughput MEAs requires scalable storage, real-time preprocessing, and efficient analytics pipelines to support continuous multimodal in vivo data streams. Without investing in cloud-enabled architectures, federated analytics, and low-latency machine learning frameworks, the transformative promise of multimodal neurodata systems will remain unfulfilled [234,235]. As these technologies advance, several significant challenges must be addressed. One of the most pressing is the suite of ethical concerns that arise with increased biological complexity and the integration of AI. These include issues of informed consent, data privacy, the speculative potential for consciousness in advanced organoid systems, and the risks of misuse in AI-powered neurotechnologies. These must be addressed proactively through robust regulatory frameworks, interdisciplinary oversight, and transparent public dialog [236,237]. In vivo experimentation remains critical for validating findings from in vitro organoid platforms and advancing translational research. However, it introduces difficulties in acquiring high-quality, artifact-free neural data, especially over extended periods and across diverse patient populations. These limitations can hinder the generalizability and long-term reliability of findings. Simultaneously, technological evolution in recording and stimulation tools has created a data deluge that current computational infrastructures struggle to manage. The need for real-time analytics, efficient storage systems, and scalable platforms is skyrocketing. Without these, the promise of integrating massive multimodal datasets into actionable insights remains unrealized.

### 5.2. Future Directions

The combination of neural interfaces with brain organoids and AI-driven systems in future research creates new possibilities, although it requires addressing major technical, biological, and ethical obstacles. The research directions aim to advance scientific knowledge by developing neurotechnologies that are ethically sound, reproducible, and human-centered. The primary path for expansion lies in combining multiple data types. Neuroengineering research necessitates the development of unified models that integrate electrophysiological recordings with imaging modalities, genomic and transcriptomic profiles, and behavioral outputs to facilitate improved interpretation. The current methods struggle to handle multi-omics data because they contain large amounts of diverse information with complex structures, which include genomics, epigenomics, proteomics, and single-cell data. AI systems need to address these challenges by creating expandable systems that integrate various data structures and generate results that reveal biological processes, rather than making predictions. The current deep learning models operate as black boxes, which creates a significant obstacle because researchers need AI frameworks that reveal biological mechanisms through interpretable results [74,234,235]. To overcome these limitations, future research needs to create deep learning systems that analyze neural and organoid data through interpretable biological frameworks to address current limitations. The implementation of Explainable AI (XAI) and Interpretable Machine Learning (IML) methods represents a promising solution because these approaches enable scientists to understand model decisions through transparent and scientifically relevant explanations. The ELI5 toolkit together with LIME (Local Interpretable Model-agnostic Explanations) and SHAP (SHapley Additive exPlanations) has shown effectiveness in medical applications for both feature importance detection and clinician decision support according to [238]. The implementation of these techniques in neuroengineering enables researchers to understand how deep learning models process neural signals, which leads to better biological interpretation and accountability. The combination of mechanistic constraints with graph-based architectures enables AI models to model neural dynamics accurately while maintaining explainability. The combination of these methods converts deep learning systems from uninterpretable prediction tools into fully transparent and dependable frameworks for advancing organoid–AI research.

The development and enhancement of organoid systems encounter multiple substantial obstacles. Traditional organoid protocols rely on speculative literature analysis and numerous rounds of in vitro testing with Matrigel and other biological substrates, resulting in poor reproducibility, limited scalability, and safety concerns regarding immunogenicity. The chemical complexity of synthetic or hybrid hydrogels requires multiple testing cycles to achieve optimal results. The process of finding suitable inducing factors and external stimuli (mechanical, electrical, optical) proves to be time-consuming and inefficient for developing standardized platforms that support organoid growth and differentiation. AI-based experimental design technology shows promise to speed up this process by determining ideal conditions that minimize biological and environmental noise between different batches [74]. The analysis of organoids through images demands new approaches. The process of extracting multiscale features from 2D/3D imaging data is time-consuming, error-prone, and heavily dependent on human judgment. The current analysis methods require manual adjustments to produce restricted measurements, which include bounding boxes; however, they cannot detect intricate details about protrusions or growth asymmetries. The development of machine learning systems that can perform precise cell- and tissue-scale morphological analysis of organoids while preserving cell movement patterns has become an immediate necessity, according to [74].

The integration of energy-based hybrid stimulation techniques into organoid–AI platforms represents a vital direction for future development. The current simulation framework requires the development of energy-based hybrid stimulation systems that unite electrical and ultrasound modalities for organoid–AI systems. The precise control of neuronal excitability through electrical stimulation occurs at low latency by using controlled current injection, but ultrasound neuromodulation provides noninvasive membrane potential and ion-channel kinetic control through acoustic radiation forces and cavitation micro effects [239]. The combination of physical stimulation methods with AI-controlled models of organoid development and neural activity will create feedback-controlled systems that use deep network predictions to generate adaptive stimulation protocols. The combination of FEM simulations with deep learning models becomes possible using physically accurate constraints for neural activation and morphological response prediction in 3D organoid environments. The optimization of stimulation parameters through reinforcement learning or variational autoencoder (VAE) controllers would produce targeted neural modulation while minimizing thermal and mechanical stress. Research has shown that machine learning systems working with ultrasound and electrical fields and multiphysics models create precise energy delivery systems for in vitro and in vivo neural activation mapping [240]. The integration of these principles into organoid–AI systems creates an opportunity to develop adaptive neurostimulation platforms that combine bioelectronic actuation with physical modeling and data-driven learning through a unified computational system.

The reliability and trustworthiness of AI models will be essential for all these domains. The central role of AI in neural decoding, stimulation, and real-time monitoring necessitates models that deliver explainable results while being ethically sound and capable of functioning across diverse population groups. The implementation of federated learning for decentralized computation and bias-mitigation strategies for underrepresented populations and XAI (Explainable AI) principles for clinical interpretability and regulatory transparency will be necessary, according to Bai et al. (2024) in their work “AI-Enabled Organoids” [74].

The quantification of behavior and affective states represents an emerging research area for applications in neurodegenerative and neuropsychiatric disorders. The next generation of closed-loop systems requires development to detect emotional and cognitive changes through wearable biosensors and real-time neural monitoring. The systems will operate through strong behavioral models that extract internal states from multiple physiological signals to generate appropriate responses. The combination of organoids with neural interface platforms will create innovative diagnostic and therapeutic tools tailored to individual patients. Organoid-based models integrated with AI and real-time recording systems will enable the prediction of disease phenotypes and drug responses before clinical symptoms appear. The successful implementation depends on achieving standardization and benchmarking across organoid manufacturing protocols, central databases, and assay pipelines to guarantee reliable translation to clinical settings.

The final requirement for all advancements demands ethical governance to lead the way. The deployment of invasive neurotechnologies and the ontological questions surrounding the sentient-like behavior of advanced organoids require public transparency, regulatory oversight, and bioethical collaboration among different fields. The development of responsible and inclusive future directions for these powerful tools requires proper frameworks that must be both equitable and thoughtful, according to [74,217–220].

## Figures and Tables

**Table 1.** Comparative overview of AI applications in early diagnosis of Alzheimer’s disease (AD) and Parkinson’s disease (PD).

AI Paradigm	Key Techniques	Applications in AD	Applications in PD	References
Machine Learning (ML)	Supervised (SVM, RF, LR); unsupervised (clustering, PCA); ensemble (bagging, boosting)	AD classification, MCI-to-AD conversion, subtype identification, feature extraction	PD classification, early-stage detection, differential diagnosis, biomarker selection	[11,52–55]
Deep Learning (DL)	DNN, CNN, RNN, autoencoders, DBN, MLP, GRU, GAN, GNN	AD/MCI identification, progression modeling, raw neuroimaging feature extraction, end-to-end classification	PD stage prediction, progression modeling, multimodal fusion, vocal biomarkers	[15,56–61]
Hybrid Models	CNN-BLSTM; AlexNet+SVM; ResNet-50+SVM; BiLSTM-ANN	Early dementia and AD detection/classification	PD detection via time-series classification, patient stratification, clinical-imaging fusion	[62–64]
Datasets Used	—	Multimodal MRI and PET, TADPOLE datasets	Structural MRI, DTI, DaTSCAN, rs-fMRI, UCI voice datasets	[65–72]

**Table 2.** Invasiveness-based classification of neural interfaces in AD and PD applications.

NI Type	Example Technologies	Description	Application in AD/PD	Pros	Cons	Sources
Invasive NIs	Utah Array, Neuralink, depth electrodes	Electrodes implanted directly in the brain/neural tissue	Used in deep brain stimulation (DBS) for PD	High-resolution, real-time brain signal access	Surgical risk, infection, long-term safety concerns, privacy, autonomy issues	[5,41,156–158]
Partially invasive NIs	ECoG grids, NeuroPace RNS	Electrodes beneath the skull but above the cortex	Experimental cognitive monitoring; seizure control	Better signal than EEG; less invasive than depth implants	Requires craniotomy; device longevity and post-surgical concerns	[159–162]
Non-invasive NIs	EEG caps, fNIRS headsets, OpenBCI, Muse, Emotiv	Scalp- or skin-surface sensors for brain activity	Cognitive and motor symptom tracking in AD/PD	Safe, affordable, widely available; home monitoring possible	Lower spatial resolution, poor depth sensing, data privacy, and algorithmic bias risks	[17,163–167]
Sensorimotor BCIs	BrainGate, Neurable, Neurocontrol Exoskeletons	Decode motor intent for control of devices or communication	Assistive devices for PD mobility or AD communication	Enables motor recovery or intent-based interaction	Cognitively demanding; limited generalization in AD; risk of over-dependence	[4,30,163,168–170]

**Table 3.** Signal-based classification of neural interfaces in AD and PD applications.

Signal Type	Example Technologies	Description	Application in AD/PD	Pros	Cons	Sources
Electrical (spikes, LFPs)	Utah Array, Neuralink, OpenBCI, ECoG, EEG	Measures electrical activity from neurons or populations	DBS, cognitive load monitoring, movement decoding in PD	High temporal resolution, real-time control	Spatial resolution varies; noise-prone (e.g., EEG)	[95,163,169]
Magnetic (MEG, OPM)	MEG, OPMs, SQUID arrays	Detects magnetic fields from neural activity without scalp contact	Cognitive monitoring, early detection of dementia-related oscillatory changes	High temporal resolution, better source localization than EEG	Expensive, bulky, limited portability	[171–174]
Optical (calcium, voltage)	Two-photon imaging, GCaMP, fNIRS, miniScope	Light-based indicators (e.g., calcium or hemoglobin changes)	Functional connectivity, early neurodegeneration detection	Cell-type specificity, good spatial resolution	Slower temporal dynamics, bulky equipment	[175–177]
Biochemical (neurotransmitters)	Neurochemical biosensors, aptamer-based electrodes	Detects levels of dopamine, glutamate, or other molecules in real time	Tracking dopamine loss in PD, identifying stress/metabolic biomarkers	High specificity to neurotransmitters	Complex fabrication, less mature than others	[178–181]
Multimodal (hybrid)	Neuralink, BrainCo, Kernel Flow, NeuroNexus	Combines electrical, optical, and metabolic signals in one platform	Comprehensive neurobehavioral assessment	Enables richer brain-state decoding	Power-hungry, complex data fusion needed	[95,104,182]
Ultrasound-based NIs	Clarity, Neural-FUS, fUSi	Ultrasound waves used for imaging (fUS) or stimulation (tFUS)	Non-invasive brain stimulation (e.g., PD) and vascular/neural imaging in AD/PD research	Deep-brain access non-invasively; high spatial resolution; diagnostic and therapeutic potential	Requires precise targeting and safety validation; limited widespread availability	[183–185]

**Table 4.** Summary of brain-specific organoid models in AD and PD research.

Organoid Model	Description	Application in AD/PD	Pros	Cons	Ethical Issues	Sources
Cerebral organoids	Mimic cortex and hippocampus for AD research	Study amyloid-beta, tau pathology (AD)	Human-relevant biology; spontaneous activity	Lack of vasculature and immune cells	Consciousness concerns in long-term culture	[23,24,28,186–189]
Midbrain organoids	Model dopaminergic neurons for PD research	Dopamine neuron loss and synaptic stress modeling (PD)	Recapitulate substantia nigra-like features	Immature and variable differentiation	Genetic manipulation implications	[190–197]
Forebrain assembloids	Fusion of forebrain and interneuron lineages	Network formation defects and migration studies in AD	Multiregional integration supports interneuron flow	Complex fabrication; reproducibility issues	Potential for sentience-like behavior	[77,198,199]
Vascularized brain organoids	Organoids embedded with vascular scaffolds	Improves AD drug diffusion, nutrient exchange modeling	Supports longer-term, functional neural development	Still under optimization; cost-intensive	Transplantation ethical risks	[129,200,201]
Fused cortico-subpallial organoids	Region-specific fused units (cortex + GABAergic)	Synaptic integration disruption analysis in AD	Enable synaptic-level interaction modeling	Requires precise fusion protocols	Moral status of fused organoids	[81,202–204]
Patient-derived iPSC organoids	Organoids from AD/PD patient stem cells	Model genetic variants in familial AD/PD	Personalized pathology, pharmacogenomic testing	Batch variability, long differentiation time	Data ownership, reprogramming rights	[205–208]
Integrated NI–organoid platforms	Electrode-organoid integration for recording/stimulation	Neurodegeneration drug screening, neuron activity tracking	Functional testing; closed-loop experiments	Still preclinical; signal interpretation challenges	Neural data privacy, AI misuse	[25,28,80,197,209]
Hippocampal organoids	Region-specific organoids mimicking the hippocampus	Memory circuit modeling, tauopathy, neurogenesis studies (AD)	Recapitulate CA- and DG-like structures; relevant to memory loss in AD	Differentiation protocols are complex; regional maturity may vary	Memory and cognition modeling raise sensitivity around sentience	[25,210–212]

## Abbreviations

The following abbreviations are used in this manuscript:

AD: Alzheimer’s Disease
AI: Artificial Intelligence
BCI: Brain–Computer Interface
BiLSTM: Bidirectional Long Short-Term Memory Network
BMI: Brain–Machine Interface
BOMA: Brain and Organoids Manifold Alignment
CNN: Convolutional Neural Network
cyb-organoid: Cyborg Organoid (hippocampal platform)
DBN: Deep Belief Network
DBS: Deep Brain Stimulation
DL: Deep Learning
DNN: Deep Neural Network
DTI: Diffusion Tensor Imaging
EC: Endothelial Cell
ECM: Extracellular Matrix
ECoG: Electrocorticography
EEG: Electroencephalography
fNIRS: Functional Near-Infrared Spectroscopy
fUS / fUSi: Functional Ultrasound Imaging
GAN: Generative Adversarial Network
GNN: Graph Neural Network
GPU: Graphics Processing Unit
GRU: Gated Recurrent Unit
hHOs: Human Hippocampal Organoids
iPSCs: Induced Pluripotent Stem Cells
LFPs: Local Field Potentials
LR: Logistic Regression
LSTM: Long Short-Term Memory Network
MDMS: Multi-Dimensional Mental States
MEAs: Microelectrode Arrays
MEG: Magnetoencephalography
ML: Machine Learning
MLP: Multi-Layer Perceptron
MPC: Metal–Polymer Conductor
mMPC: Mesh Metal–Polymer Conductor
MRI: Magnetic Resonance Imaging
ND: Neurodegenerative Diseases
NEURON: Simulation environment for modeling neurons and networks
OBCI: Organoid–Brain–Computer Interface
OPM: Optically Pumped Magnetometer
PCA: Principal Component Analysis
PD: Parkinson’s Disease
PET: Positron Emission Tomography
RNN: Recurrent Neural Network
RF: Random Forest
rs-fMRI: Resting-State Functional Magnetic Resonance Imaging
SQUID: Superconducting Quantum Interference Device
SRC: Signal-Receiving Cell
SSC: Signal-Sending Cell
SVM: Support Vector Machine
TADPOLE: Alzheimer’s Disease Prediction Challenge Dataset
tFUS: Transcranial Focused Ultrasound Stimulation
UCI: University of California Irvine (voice datasets)
XAI: Explainable Artificial Intelligence
